# Antioxidant properties, antimicrobial and anti-adhesive activities of DCS1 lipopeptides from *Bacillus methylotrophicus* DCS1

**DOI:** 10.1186/s12866-017-1050-2

**Published:** 2017-06-28

**Authors:** Nawel Jemil, Hanen Ben Ayed, Angeles Manresa, Moncef Nasri, Noomen Hmidet

**Affiliations:** 1 Laboratoire de Génie Enzymatique et de Microbiologie, Université de Sfax, Ecole Nationale d’Ingénieurs de Sfax, B.P, 1173-3038 Sfax, Tunisia; 2 Section of Microbiology, Department of Biology, Health and Environment, Faculty of Pharmacy, University of Barcelona, Joan XXIII s/n, 08028 Barcelona, Spain

**Keywords:** *Bacillus methylotrophicus* DCS1, Lipopeptides like substances, Antioxidant, Antimicrobial, Anti-adhesive activities

## Abstract

**Background:**

The present work aims to investigate the antioxidant and antimicrobial activities as well as the potential of DCS1 lipopeptides produced by *Bacillus methylotrophicus* DCS1 strain at inhibition and disruption of biofilm formation.

**Results:**

The produced biosurfactants were characterized as lipopeptides molecules by using thin layer chromatography (TLC) and Fourier transform infrared spectroscopy (FT-IR). The DCS1 lipopeptides were assayed for their antioxidant activity through five different tests. The scavenging effect on DPPH radicals at a concentration of 1 mg mL^−1^ was 80.6%. The reducing power reached a maximum value of 3.0 (OD_700 nm_) at 2 mg mL^−1^. Moreover, the DCS1 lipopeptides exhibited a strong inhibition of β-carotene bleaching by linoleic acid assay with 80.8% at 1 mg mL^−1^ and showed good chelating ability and lipid peroxidation inhibition. The in vitro antimicrobial activity of DCS1 lipopeptides showed that they display significant antibacterial and antifungal activities. The anti-adhesive activity of DCS1 lipopeptides was evaluated against several pathogenic microorganisms. The lipopeptides showed excellent anti-adhesive activity, even at low concentrations, in a polystyrene surface pre-treatment against all the microorganisms tested. Further, they can disrupt performed biofilms.

**Conclusion:**

This study shows the potentiality of DCS1 lipopeptides as natural antioxidants, antimicrobial and/or anti-adhesive agent for several biomedical and industrial applications.

## Background

Bacteria are the main group of biosurfactants producing microorganisms [1]. Several studies have reported the potential of *Bacillus* species as biosurfactant producers such as lipopeptide type biosurfactants [2]. These are amphiphilic cyclic peptides that are linked to a fatty acid hydrocarbon chain and they belong to the surfactin, iturin and fengycin families. They are synthesized by non-ribosomal peptide synthetases without involving messenger RNA [3]. Lipopeptides were found to have specific biological activities, such as antioxidant [4, 5], antimicrobial [6–8], and antitumor activities [9]. The advantages related to the use of microbial biosurfactants over their chemical counterparts include their lower toxicity and higher biodegradability, as well as their effectiveness at different environmental conditions such as extreme pH, temperature and high ionic strength, in addition to their biocompatibility [10]. These advantages allow using them in pharmaceutical, cosmetic and food additives industries [11]. In the food industry, biosurfactants offer several functions such as emulsifying/foaming agents, stabilizers, antioxidant agents, and anti-adhesives [12, 13].

Biosurfactants have also been proved to be great inhibitors of microbial adhesion and biofilm formation. Biofilms are communities of microbes adhering to biotic or abiotic surfaces. Broadly, microbial biofilms are a daily challenge faced by the food industry and society [14]. Adhesion is the first stage of biofilm formation and the best time for the action of anti-adhesive compounds [15]. In fact, life in a biofilm probably represents the predominate mode of growth for microbes in most environments. The role of biosurfactants in microbial anti-adhesion and desorption has been widely described, and adsorption of biosurfactants to solid surfaces can be an effective strategy to reduce microbial adhesion and combating colonization by pathogenic microorganisms [16, 17].

This report represents an investigation of the antioxidant, antimicrobial and anti-adhesive activities of lipopeptides produced by *Bacillus methylotrophicus* DCS1 strain.

## Methods

### Microorganism

Biosurfactant-producing strain used in this study was isolated from hydrocarbon contaminated soil in Sfax City, Tunisia. It was selected on the basis of the high hemolytic activity and decreasing surface tension of the culture medium from 72 mN m^−1^ to 31 mN m^−1^. This strain was identified as *Bacillus methylotrophicus* DCS1 based on its biochemical and 16S rDNA gene sequence analysis [18]. The bacterial strain was maintained at 4 °C and also preserved in glycerol at −80 °C.

### Production media

*Bacillus methylotrophicus* DCS1 was inoculated into a 250 mL Erlenmeyer flask containing 25 mL Luria-Bertani (LB) broth medium (g L^−1^): peptone, 10.0; yeast extract, 5.0; and NaCl, 5.0; pH 7.0. Culture was incubated at 37 °C with shaking at 200 rpm for 18 h. A 3% (*v*/v) of inoculum [OD_600 nm_ = 7.6] was transferred into a 2 L Erlenmeyer flask containing 250 mL of Landy medium [19] which contains: glucose 20 g L^−1^, L-glutamic acid 5 g L^−1^, yeast extract 1 g L^−1^, K_2_HPO_4_ 1 g L^−1^, MgSO_4_ 0.5 g L^−1^, KCl 0.5 g L^−1^, CuSO_4_ 0.0016 g L^−1^, Fe_2_ (SO_4_)_3_ 0.0004 g L^−1^, MnSO_4_ 0.0012 g L^−1^. The initial pH of the medium was adjusted to 7.0 and culture was incubated for 72 h at 30 °C with shaking at 150 rpm.

### Biosurfactant recovery

The culture medium was centrifuged at 8000 rpm and 4 °C for 20 min to remove bacterial cells. Supernatant free-cell was subjected to acid precipitation with 6 N HCl until pH 2.0, then left overnight at 4 °C with agitation. The precipitate was collected by centrifugation at 8000 rpm, for 20 min at 4 °C, suspended in distilled water and the pH was adjusted to 8.0 with NaOH (1.0 N) [20]. The crude biosurfactants obtained was lyophilized.

### Thin layer chromatography

The extracted biosurfactants were analyzed on silica plates 60 F (Merck, Macherel-Nagel) with a solvent system consisting of (chloroform: methanol: water) solution in the ratio (65:25:4). The compounds on the plate were detected after spraying with the ninhydrin reagent and iodine vapor which respectively detected the peptide and lipid parts in the biosurfactants [21].

### FT-IR spectra of the dried biosurfactants

Fourier transform infrared spectroscopy (FT-IR) was used to determine the functional groups and the chemical bonds present in the biosurfactants extract and then to determine its chemical nature. The FT-IR analysis was realized by using Analect Instruments fx-6160 FT-IR spectrometer. 1 mg of lyophilized sample was ground in about 100 mg of spectral grade KBr and pressed for 30 s to obtain translucent pellets. The spectral measurements were carried out in the absorbance mode. The FTIR spectrum of the prepared biosurfactants was recorded between 400 and 4000 cm^−1^.

### Antioxidant activities

#### DPPH radical-scavenging assay

The 2, 2-diphenyl-1-picrylhydrazyl (DPPH) free radical-scavenging potential of DCS1 lipopeptides at different concentrations (0.1–2 mg mL^−1^) was determined according to the report of Bersuder et al. [22]. The DPPH radical scavenging activity was calculated as follows:

$$ \mathrm{DPPH}\ \mathrm{radical}\hbox{-} \mathrm{scavenging}\ \mathrm{activity}\ \left(\%\right)=\frac{{\mathrm{A}}_{\mathrm{control}}+{\mathrm{A}}_{\mathrm{blanc}}-{\mathrm{A}}_{\mathrm{sample}}}{{\mathrm{A}}_{\mathrm{control}}}\times 100 $$

where A_control_ is the absorbance of the control reaction (containing all reagents except lipopeptides), A_blank_ is the absorbance of lipopeptides (containing all reagents except the DPPH solution) and A_sample_ is the absorbance of lipopeptides with the DPPH solution.

Lower absorbance of the reaction mixture indicated higher DPPH radical-scavenging activity. BHA (2.0 mM) was used as positive standard. The test was carried out in triplicate and the results are mean values.

The effective concentration of DCS1 lipopeptides required to scavenge DPPH radical by 50% (IC_50_ value) was obtained by linear regression analysis of dose-response curve plotting between percentage of DPPH radical-scavenging activity and concentrations.

### Ferric-reducing activity

The ability of DCS1 lipopeptides at different concentrations (0.1 to 3 mg mL^−1^) to reduce iron III was determined according to the method of Yildirim et al. [23]. All data values presented are the mean of triplicate analyses.

### Ferrous ion-chelating activity

The Fe^2+^ chelating activity of DCS1 lipopeptides at different concentrations (0.5–5 mg mL^−1^) was estimated by the method of Carter [24]. The percentage of inhibition of ferrozine–Fe^2+^ complex formation was calculated using the following formula:

$$ \mathrm{Ferrous}\ \mathrm{ion}\hbox{-} \mathrm{chelating}\ \mathrm{activity}\ \left(\%\right)=\frac{{\mathrm{A}}_{\mathrm{control}}+{\mathrm{A}}_{\mathrm{blanc}}-{\mathrm{A}}_{\mathrm{sample}}}{{\mathrm{A}}_{\mathrm{control}}}\times 100 $$

where A_control_ is the absorbance of the control (without sample), A_blank_ is the absorbance of the blank (without ferrozine) and A_sample_ is the absorbance of the sample. Ethylene diamine tetra acetic acid (EDTA) was used as positive control and the test was carried out in triplicate.

### β-carotene bleaching assay

The capacity of DCS1 lipopeptides to prevent bleaching of β-carotene at different concentrations (0.025–1 mg mL^−1^) was evaluated as described by Koleva et al. [25]. The antioxidant activity was calculated in terms of β-carotene bleaching inhibition using the following formula:

$$ \upbeta \hbox{-} \mathrm{carotene}\ \mathrm{bleaching}\ \mathrm{inhibition}=\left[1-\left(\frac{{\mathrm{A}}_0-{\mathrm{A}}_{\mathrm{t}}}{{{\mathrm{A}}^{\hbox{'}}}_0-{{\mathrm{A}}^{\hbox{'}}}_{\mathrm{t}}}\right)\right]\times 100 $$

where A_0_ and A’_0_ are the absorbances of the test sample and the control, respectively, measured at time zero; and A_t_ and A’_t_ are the absorbance of the sample and the control, respectively, measured after incubation. BHA was used as a positive standard. Tests were performed in triplicate and values presented are the mean of triplicate analyses.

The half maximal inhibitory concentration (IC_50_) is the concentration where 50% inhibition of the β-carotene bleaching radical is obtained. IC_50_ value was obtained by linear regression analysis of dose-response curve plotting between percentage of inhibition and concentrations.

### Inhibition of linoleic acid peroxidation

In vitro lipid peroxidation inhibitory activity of lipopeptides like substances DCS1 was determined by evaluating their ability to inhibit oxidation of linoleic acid in an emulsified model system. DCS1 lipopeptides at 0.1 mg mL^−1^ concentration were dissolved in 2.5 mL of distilled water and added to 2.5 mL of ethanol and 32.5 μL linoleic acid. The final volume was then adjusted to 6.25 mL with distilled water. The mixture was incubated in a 10 mL tube with silicone rubber caps at 45 °C for 9 days in dark. Vitamin C, a natural antioxidant agent, was used as a positive control, and distilled water as negative control. Lipid peroxidation was estimated as evidenced by the formation of thiobarbituric acid reactive substances (TBARS) such as malondialdehyde (MDA). TBARS were tested in samples by the method described by Yagi [26]. MDA and other TBARS were measured by their reactivity with TBA in an acidic condition to generate pink colored chromospheres which was read at 530 nm. A sample (375 μL) was homogenized with 150 μL of TBS (50 mM Tris containing 150 mM NaCl, pH 7.4) and 375 μL of TCA 20% (*w*/w) in order to precipitate proteins, and then centrifuged (1000 g, 10 min, 4 °C). A 400 μL of the supernatant was mixed with 80 μL of HCl (0.6 M) and 320 μL of Tris–TBA (Tris 26 mM; TBA 120 mM), and the mixture was heated for 10 min at 80 °C. The absorbance of the resulting solution was read at 530 nm. Inhibition of linoleic acid peroxidation was estimated by the following formula:

$$ \mathrm{Inhibition}\ \mathrm{of}\ \mathrm{linoleic}\ \mathrm{acid}\ \mathrm{peroxidation}=\left[1-\frac{{\mathrm{A}}_{\mathrm{sample}}}{{\mathrm{A}}_{\mathrm{control}}}\right]\times 100 $$

where A_sample_ was the absorbance of the sample and A_control_ was the absorbance of the control (replacing the sample by water).

### Antimicrobial activities

#### Microbial strains

Antibacterial activity of DCS1 lipopeptides was tested against three Gram-positive bacteria: *Staphylococcus aureus* (ATCC 25923), *Bacillus cereus* (ATCC 11778), *Micrococcus luteus* (ATCC 4698) and five Gram-negative bacteria: *Klebsiella pneumoniae* (ATCC 13883), *Escherichia coli* (ATCC 25922), *Salmonella typhimurium* (ATCC 19430), *Salmonella enterica* (ATCC 27853) and *Enterobacterium* sp.

Antifungal activity was tested using *Aspergillus niger*, *Aspergillus flavus*, *Fusarium oxysporium*, *Pythium ultiumum*, *Fusarium solani* and *Rhizoctonia bataticola*.

### Agar diffusion method

DCS1 lipopeptides were tested in vitro for antimicrobial activities by conventional agar diffusion method against human pathogens [27].

Using sterile pipette, culture suspension (200 μL) of the tested microorganisms (10^6^ colony-forming units CFU mL^−1^) of bacteria cells (estimated by absorbance at 600 nm) and 10^8^ spores mL^−1^ of fungal strains (measured by Malassez blade) were spread uniformly on Luria-Bertani agar and malt extract agar media, respectively. Then, wells were made using a sterile well borer and were filled with 100 μL of lipopeptides sample (2 mg mL^−1^ concentration).

The zone of growth inhibition was measured in millimetres after incubation for 24 h at 37 °C for bacteria and for 72 h at 30 °C for fungal strains. All the results were represented as the average of three independent experiments.

### Effect of proteolytic enzymes, heat, and pH on crude lipopeptides antibacterial activity

The susceptibility of the crude lipopeptides to proteolytic enzymes, heat and pH treatments was assessed as described elsewhere [28]. To evaluate its stability to proteolytic enzymes, the crude lipopeptides sample (2 mg mL^−1^ concentration) was incubated at 37 °C for 60 min with 1 mg mL^−1^ final concentration of trypsin, chymotrypsin and pepsine. To analyze thermal stability, samples were incubated at temperatures ranging 40, 60, 80 and 100 °C for 20 min. The effect of pH on DCS1 lipopeptides activity was studied by assaying antibacterial activity against *K. pneumoniae*, the pH was adjusted at values from 3.0 to 10.0. Samples were neutralized to pH 7.0 before measurement of the antibacterial activity, the following buffer systems were used: 100 mM glycine–HCl buffer, pH 3.0–4.0; 100 mM acetate buffer, pH 4.0–6.0; 100 mM Tris–HCl buffer, pH 7.0–8.0 and 100 mM glycine–NaOH buffer, pH 9.0–10.0. After the treatments, residual antibacterial activity against *K. pneumoniae* was determined as follows:

$$ \mathrm{Residual}\ \mathrm{antibacterial}\ \mathrm{activity}\ \left(\%\right)=\frac{\mathrm{Zone}\ \mathrm{of}\ \mathrm{growth}\ \mathrm{inhibition}\ \mathrm{after}\ \mathrm{treatment}}{\mathrm{Zone}\ \mathrm{of}\ \mathrm{growth}\ \mathrm{inhibition}\ \mathrm{without}\ \mathrm{treatment}}\times 100 $$

### Anti-adhesion treatment with lipopeptides extract DCS1

The anti- and post-adhesion treatments were studied with lipopeptides extract obtained as follows: acid-precipitated lipopeptides (1 g) was subjected to extraction with 25 ml tetrahydrofuran (THF) solvent four times and the mixture was stirred and centrifuged at 8000 rpm, for 15 min at 4 °C. The organic phases recuperated were combined and evaporated to dryness in a rotary vacuum evaporator (Büchi, Switzerland) at 40 °C, and then lipopeptides extract was suspended in distilled water and lyophilized.

For surface pre-treatment, the wells of a sterile micro-titer plate were loaded with 200 μL of DCS1 lipopeptides extract at different concentrations ranging from 0.016 to 2 mg mL^−1^, dissolved in PBS (pH 7.2). Micro-titer plates were incubated for 6 h at room temperature (25 °C) and then washed twice with PBS.

For biofilm formation, *Salmonella typhimurium* ATCC 14028, *Klebsiella pneumoniae* ATCC 13883, *Staphylococcus aureus* ATCC 25923, *Bacillus cereus* ATCC 11778 and *Candida albicans* ATCC 10231 were cultured overnight in Luria-Bertani medium (LB). Cultures were diluted 1/100 in the medium proposed by O′Toole [29] (g L^−1^): glucose, 2; casamino acids, 5; KH_2_PO_4_, 3; K_2_HPO_4_, 7; (NH_4_)_2_SO_4_, 2, MgSO_4_ 7H_2_O, 0.12. Then, 200 μL of each dilution were added to the micro-titer plate wells and incubated for 20 h at 37 °C. After incubation, wells were washed three times with distilled water to remove non-adherent cells, fixed for 15 min with methanol and stained with 125 μL crystal violet (CV) (0.1%) for 20 min, then washed with water and dryed. For quantifying the microbial adhesion, the stain in the wells was diluted with 200 μL acetic acid in water (33%) and the absorbance was determined at 595 nm [13]. Percentages of microbial adhesion inhibition were calculated using the formula:

$$ \mathrm{Microbial}\ \mathrm{adhesion}\ \mathrm{inhibition}=\left[1{\textstyle \hbox{-}}\left({\mathrm{A}}_{\mathrm{c}}/{\mathrm{A}}_0\right)\operatorname{}\right]\times 100 $$

where A_c_ represents the absorbance of the well with lipopeptides at concentration c and A_0_ represents the absorbance of the positive control wells (in absence of lipopeptides). Negative control wells contained only lipopeptides dissolved in PBS. Assays were carried out three times.

### Mature biofilm treatment with lipopeptides extract DCS1

The wells of a polystyrene micro-titer plates were filled with 200 μL of bacterial suspension prepared as described above, and then the plates were incubated for 20 h at 37 °C. After incubation, the unattached microbial cells were removed by washing the wells three times with distilled water. Thereafter, 200 μL of DCS1 lipopeptides at different concentrations ranging from 0.016 to 2 mg mL^−1^, were added to each well and the plates were incubated for 6 h at room temperature (25 °C). The quantification was realized as in the pre-treatment. All the results were represented as the average of three independent experiments.

All data presented are the average of at least three measurements which deviated by not more than 5%.

## Results and discussion

### Preliminary chemical characterization

TLC analysis separates compounds in a mixture and can be used to determine the number of components in solutions. The sample of crude biosurfactants revealed yellow spots with iodine vapour, suggesting the presence of polar lipids. Treatment with ninhydrin revealed pink spots suggesting the presence of protein portions. The presence of both protein units and lipid moieties on the same spot suggested that the sample is a lipopeptide type. Similar results for other lipopeptide type biosurfactant were also reported elsewhere [30].

**Fig. 1:**
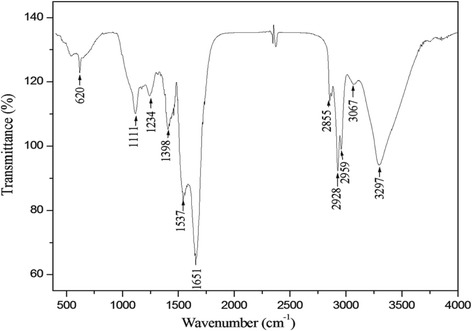
Fourier transforms infrared spectrum of biosurfactants produced by *Bacillus methylotrophicus* DCS1

The IR spectrum of the crude biosurfactants DCS1 was analyzed to gain insight into its chemical nature (Fig. 1). A broad absorbance with wave numbers ranging approximately from 3700 cm^−1^ to 3000 cm^−1^ having its maximum at 3297 cm^−1^ was detected. Absorbance in this region is a result of -CH and -NH stretching vibrations, and it is a characteristic of carbon-containing compounds with amino groups [31]. Other sharp absorbance peaks are seen at 2959, 2928 and 2855 cm^−1^, indicating the presence of –C-CH_3_ banding or long alkyl chains [32]. The peak with highest absorbance in the spectrum was observed at 1651 cm^−1^. Absorbance in this region signifies the presence of peptide groups in the molecules [33]. Another high intensity peak at 1537 cm^−1^, corresponded to the deformed N–H band. The weak band at 1398 cm^−1^ in the absorption range 1370–1470 cm^−1^ could result from deformation and bending vibrations of –C-CH_2_ and –C-CH_3_ groups in aliphatic chains [34]. Peaks at 1234 and 1111 cm^−1^ are probably due to the presence of C-O-C vibrations in esters [32, 33].

The observed peaks are those commonly found in the IR spectra of lipopeptide biosurfactants produced by several species [21, 35, 36].

### Antioxidant activities

### DPPH radical-scavenging assay

**Fig. 2:**
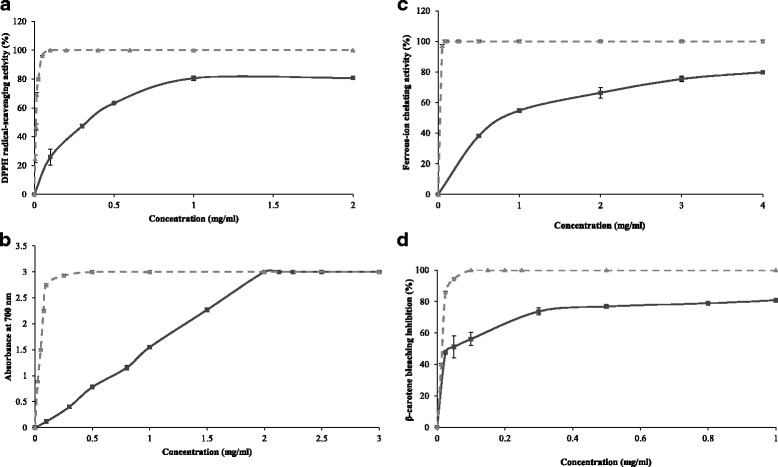
Scavenging effect on DPPH free radical (**a**), ferric-reducing antioxidant power (**b**), ferrous-ion chelating activity (**c**) and β-carotene bleaching assay (**d**) of DCS1 lipopeptides at different concentrations. BHA and EDTA (2.0 mM) were used as positive control. Values presented are the mean of triplicate analysis.  Lipopeptides DCS1  BHA  EDTA

The DPPH radical-scavenging assay has been generally used to study the ability of compounds to act as free radical scavengers or hydrogen donors [37]. When DPPH encounters a hydrogen-donating substance, the radical would be scavenged, as visualized by changing its color from purple to yellow, and the absorbance is reduced [38]. The stable DPPH radical displays a maximum absorbance at 517 nm in ethanol. As displayed in Fig. 2a, the DCS1 lipopeptides exhibited effective antioxidant activity against DPPH in a dose dependent manner. In fact, at 1 mg mL^−1^, the DCS1 lipopeptides showed a potential scavenging effect of 80.6%, which is three-times higher than that obtained at 0.1 mg mL^−1^ (25.9%). Our results are in accordance with previous works of Yalçin and Ҫavuşoǧlu [39] who reported that the DPPH scavenging activity increased with increasing the concentration of biosurfactant synthesized by *Bacillus subtilis* RW-I.

However, DCS1 lipopeptides exhibited lower radical-scavenging activity than did BHA used as reference at the same concentrations. The IC_50_ value was about 357 μg mL^−1^. Scavenging effect can be assigned to the presence of the hydrocarbon fatty acid chain and some active residues in the peptide ring, which acted as a good hydrogen atom or an electron donor and could react with free radicals of DPPH.

#### Ferric reducing antioxidant power

The reducing power of a compound may serve as a significant indicator of its potential antioxidant activity [40]. In this test, the presence of reducers causes the reduction of the Fe^3+^/ferricyanide complex to the ferrous form. The reductive activity of DCS1 lipopeptides as well as the synthetic antioxidant BHA as a function of their concentrations is illustrated in Fig. 2b. The reducing capacity of DCS1 lipopeptides increased with increasing their concentration. It indicates a dose-dependent response and reached a maximum of 3.0 (OD_700 nm_) at a concentration of 2.0 mg mL^−1^. The obtained values are lower than those of BHA at concentrations below 2.0 mg mL^−1^.

The reductive ability of DCS1 lipopeptides is higher than those of lipopeptides produced by *Bacillus mojavensis* A21 which reached 2.0 (OD_700 nm_) at a concentration of 10 mg mL^−1^ [5] and surfactin lipopeptide produced by *B. subtilis* RW-I, which reached 2.0 (OD_700 nm_) at a concentration of 2.5 mg mL^−1^ [39]. Yalçin and Ҫavuşoǧlu [39] reported that the reductive ability could be related to the presence of hydroxyl groups in the lipopeptides molecules.

### Ferrous ion-chelating activity

Ferrous ion (Fe^2+^) is the most potent pro-oxidant among metal ions. This ion can interact with hydrogen peroxide in a Fenton reaction to produce the reactive oxygen species and hydroxyl free radical (OH), leading to the initiation and/or acceleration of lipid oxidation [41]. Chelating agents may inhibit lipid oxidation by stabilizing transition metals. Measurement of color reduction allows, therefore, estimating the metal chelating activity of the co-existing chelator [42].

The Fe^2+^ chelating capacity of DCS1 lipopeptides against Fe^2+^ was determined by measuring the iron ferrozine complex. As shown in Fig. 2c, DCS1 lipopeptides exhibited strong ferrous-chelating activity and chelated almost 79.8% of ferrous ions at 4 mg mL^−1^. However, DCS1 lipopeptides exhibited lower metal chelating activity than EDTA, a well-known metal ion chelator. The obtained results suggest that some lipopeptide isoforms could be potential antioxidant through metal chelating ability.

### β-carotene bleaching assay

The antioxidant assay using the discoloration of β-carotene is widely used to measure the antioxidant activity of bioactive compounds. The β-carotene bleaching inhibition was tested at different concentrations and compared with BHA. As shown in Fig. 2d, the DCS1 lipopeptides inhibited significantly the discoloration of β-carotene and its antioxidant activity increased with increasing sample concentration. The inhibitor concentration IC_50_ was estimated to be 42 μg mL^−1^. However BHA has better β-carotene bleaching inhibition than that of DCS1 lipopeptides at all concentrations tested. DCS1 lipopeptides are more effectives than A21 lipopeptides, which showed a β-carotene bleaching inhibition of 72% at 10 mg mL^−1^ concentration and the IC_50_ was estimated to be 3.7 mg mL^−1^ [5].

### Inhibition of linoleic acid peroxidation

**Fig. 3:**
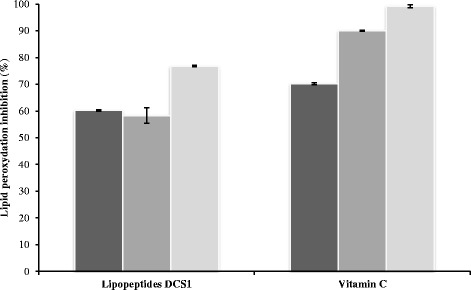
Inhibition of linoleic acid peroxidation by DCS1 lipopeptides. 3 days 6 days 9 days

Antioxidant activity of DCS1 lipopeptides, at a concentration of 0.1 mg mL^−1^, against the peroxidation of linoleic acid during 3, 6 and 9 days of storage at 45 °C, was evaluated and compared to that of vitamin C, used as a natural antioxidant (Fig. 3). After 3 days of incubation period, DCS1 lipopeptides displayed a lipid peroxidation inhibition of about 60.22%, and reached about 76.8% after 9 days. The vitamin C showed higher protective effect than biosurfactants. The inhibitory effect could be explained by the presence of acyl chain of fatty acids by improving the interaction between lipopeptide like substances and linoleic acid [5]. The crude lipopeptides synthesized by *B. methylotrophicus* DCS1 strain can be an important potential source of antioxidants.

### Antimicrobial activity of DCS1 lipopeptides

The antimicrobial activity of DCS1 lipopeptides, against various microorganisms, was estimated by agar well diffusion method and the results are summarized in Table 1. Lipopeptides were effective against several tested bacteria with different degrees. The highest antibacterial activity was observed against *K. pneumoniae*, with a maximum zone diameter inhibition of 30 mm at a concentration of 2 mg mL^−1^, while the lowest activity was detected against *Enterobacterium* sp. However, DCS1 lipopeptides did not exhibit antimicrobial activity against *M. luteus* at 2 mg mL^−1^. Several lipopeptide biosurfactants obtained from *Bacillus amyloliquefaciens* M1 [8] and *Bacillus mojavensis* A21 [7] showed strong antibacterial activity.

**Table 1:** Antimicrobial activity spectrum of DCS1 lipopeptides (2 mg mL^−1^)

Indicator organisms		Inhibition zone diameter (mm)
Gram (+)		
*S. aureus* (ATCC 25923)		14 ± 0.4
*B. cereus* (ATCC 11778)		15 ± 0.1
*M. luteus* (ATCC 4698)		−
Gram (−)		
*K. pneumoniae* (ATCC 13883)		30 ± 0.1
*E. coli* (ATCC 25922)		16.5 ± 0.6
*S. typhimyrium* (ATCC 19430)		15 ± 0.2
*S. enterica* (ATCC 27853)		15 ± 0.1
*Enterobacterium* sp.		7.66 ± 0.6
Fungi		
*A. niger*		+++
*A. flavus*		+++
*F. oxysporium*		−
*P. ultimum*		++
*F. solani*		++
*R. bataticola*		++

Determinations were performed in triplicate and data correspond to mean values ± standard deviations

Regarding the antifungal activity, DCS1 lipopeptides showed a significant activity against various fungal strains. Higher activity was observed against *A. niger* and *A. flavus*, less activity was against *P. ultimum*, *F. solani* and *R. bataticola*, no effect was observed on *F. oxysporium*. Donio et al. [43] reported that *Bacillus* sp. BS3’s biosurfactants possess an important antifungal activity against different pathogens. Sheppard et al. [44] indicated that lipopeptide biosurfactants interact with the cell membrane of the target cells and exhibit significant antimicrobial properties.

### Effect of proteolytic enzymes, heat and pH on lipopeptides antibacterial activity

The resistance of lipopeptides against proteases and extreme conditions, including temperature and pH was a prerequisite for their potential therapeutic and pharmaceutical applications. DCS1 lipopeptides retained their antibacterial activity against *K. pneumoniae* after incubation for 1 h with proteolytic enzymes pepsin, trypsin and chymotrypsin at a concentration of 1 mg mL^−1^ (Table 2). These results exclude the eventual existence of bacteriocin-like substances in the crude lipopeptides and they indicate that the antimicrobial compounds could be cyclic peptides containing unusual amino acids [45].

**Table 2:** Influence of proteolytic enzymes, temperature and pH on DCS1 lipopeptides antibacterial activity against *K. pneumoniae*

Treatment	Residual activity (%)
Enzyme (1 mg mL^−1^)	
Pepsin	100
Trypsin	100
Chymotrypsin	100
Temperature (°C)	
40	100
60	100
80	100
100	100
pH	
3	74
5	84
8	100
10	100

Residual activity compared with the antimicrobial activity before the treatment. Data are means of three independent experiments

Regarding thermostability, DCS1 lipopeptides were resistant to heating for 20 min at temperatures up to 100 °C retaining 100% their initial activity (Table 2). The maintenance of antimicrobial activity after treatment with proteolytic enzymes and after incubation at a wide range of temperature resembles the characteristics of cyclic lipopeptides of *Bacillus* sp. [46].

Antimicrobial activity was also tested at different pH values. The antimicrobial substance was active at pH 8.0 and pH 10.0, retaining 100% of the activity, while at pH 5.0 and 3.0 the activity was decreased (84.4% and 74% of its initial activity at pH 5.0 and pH 3.0, respectively). The decrease in antibacterial activity could be due to partial precipitation of lipopeptides.

### Anti-adhesive activity

**Fig. 4:**
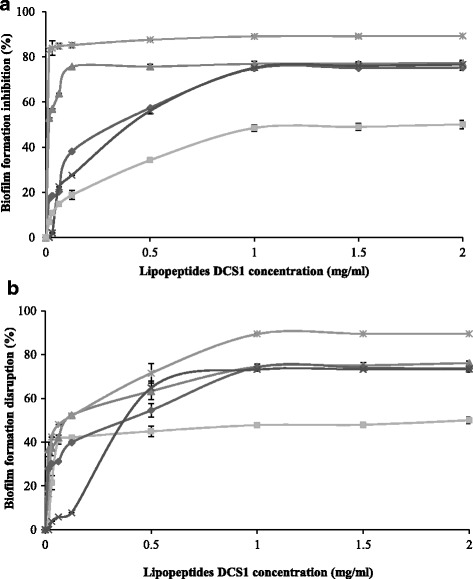
Effect of DCS1 lipopeptides at different concentrations on biofilm formation inhibition (**a**) and disruption (**b**).  *Staphylococcus aureus*  *Salmonella typhimurium*  *Klebsiella pneumoniae*  *Candida albicans*  *Bacillus cereus*

Biosurfactants are known to decrease the adhesion of pathogenic microorganisms to solid surfaces [47]. Thus, the anti-adhesive activity of DCS1 lipopeptides was investigated against five strains. As can be seen in Fig. 4a, the pre-treatment of polystyrene surfaces with DCS1 lipopeptides significantly inhibited the adhesion of all tested bacteria, even at low concentrations. The biofilm formation inhibition was concentration-dependent, and the anti-adhesive effect remains nearly constant above a concentration of 1 mg mL^−1^ lipopeptides. The highest anti-adhesive effect was observed against *C. albicans* with an inhibition percentage of about 89.3%. Our results are in accordance with those of Janek et al. [15] who reported that the pre-treatment of a polystyrene surface with 0.5 mg mL^−1^ pseudofactin II inhibited bacterial adhesion by 36–90% and that of *C. albicans* by 92%. In another study, Coronel-león et al. [13] reported that the highest anti-adhesive activity of the biosurfactant lichenysin produced by *Bacillus licheniformis* AL1.1 was observed against *C. albicans* (74.35%) at a concentration of 4 mg mL^−1^.

High anti-adhesion effect was also observed for *S. aureus*, *B. cereus* and *S. typhimurium* (77.3, 77.1 and 75.7%, respectively, at 1 mg mL^−1^) with DCS1 lipopeptides, while the effect on *K. pneumoniae* was low (50%). Gudiña et al. [47] demonstrated high anti-adhesion activity against many pathogens, among them *S. aureus* (72.0%), *Staphylococcus epidermidis* (62.1%) and *Sreptococcus agalactiae* (60%), but at high concentration of biosurfactant produced by *Lactobacillus paracasei* A20 (25 mg mL^−1^). Earlier reports [48] described the inhibition of pathogenic bacteria adhesion (*Escherichia coli* CFT073 and *S. aureus* ATCC 29213) to polystyrene surfaces by two lipopeptide biosurfactants synthesized by *Bacillus subtilis* and *Bacillus licheniformis*. Furthermore, *Pontibacter korlensis* SBK-47 strain produces pontifactin, a new lipopeptide biosurfactant that exhibits anti-adhesive activity against many pathogenic bacteria ranging from 87% to 99% inhibition at a maximum concentration of 2 mg mL^−1^ [36].

DCS1 lipopeptides exhibit high efficiency as the calculated effective dose (ED_50_) with 50% adhesion inhibition was very low with all microorganisms tested: 0.36 mg mL^−1^ for *S. typhimurium*, 2 mg mL^−1^ for *K. pneumoniae*, 0.4 mg mL^−1^ for *B. cereus* and 0.015 mg mL^−1^ for *S. aureus*.

The inhibition of biofilm formation is due to the capacity of lipopeptides to modify the physico-chemical properties of the surface to reduce adhesion and biofilm formation. Some lipopeptides are considered anionic due to the negative charges of amino acids, also surfaces of most bacterial cells are negatively charged. Therefore, in the pre-treatment with lipopeptides, the potential effect of biofilm formation inhibition could be the result of the electrostatic repulsion forces between the negative charge of polystyrene surface recovered with lipopeptides molecules and negative charge of the microbial membrane. Bacterial adhesion depends on bacterial charge, surface type and on the biosurfactant charge which interferes in their influences.

### Disruptive activity on pre-formed biofilm

Disruption of biofilm consists in removing the attached microorganisms from the surface after biofilm formation. As illustrated in Fig. 4b, the efficacy of biofilm disruption increased with increasing sample concentration, and the percentages of disruption remain nearly constant above a concentration of 1 mg mL^−1^ lipopeptides. The maximum disruption produced by DCS1 lipopeptides was observed for *C. albicans* with 89.50%, followed by *S. aureus* (77%), *S. typhimurium* (75%), *B. cereus* (74.8%) and *K. pneumoniae* (50.3%). Our results are in agreement with those of Dalili et al. [12] who reported that Coryxin, a cyclic lipopeptide, produced by *Corynebacterium xerosis* NS5 displayed inhibitory and disruptive activities against biofilm formation by a variety of bacteria.

The effectiveness of lipopeptides in post-treatment of biofilm formation using various microorganisms was similar to that in pre-treatment. Our findings are in contrast with those of Janek et al. [15] who stated that the effectiveness in biofilm disruption in a post-treatment is lower than in biofilm inhibition in a pre-treatment with an activity ranging from 26 to 70% with pseudofactin II (0.5 mg mL^−1^).

The effective dose (ED_50_) was very low in post-treatment with all pathogens tested: 0.51 mg mL^−1^ for *S. typhimurium*, 2 mg mL^−1^ for *K. pneumoniae*, 0.11 mg mL^−1^ for *S. aureus*, 0.37 mg mL^−1^ for *B. cereus* and 0.096 mg mL^−1^ for *C. albicans*.

The disruption of biofilm could be explained by the adsorption of lipopeptides at the interface between the attached biofilm-forming bacteria and the solid surface, thus favoring bacterial detachment. According to McLandsborough et al. [49], surfactants could penetrate and adsorb at the interface between the solid surface and the biofilm owing to their high surface activity and reduce the interfacial tension.

When comparing both processes, pre-treatment and post-treatment of polystyrene surfaces, it is clear that the action of DCS1 lipopeptides was more effective against *C. albicans*. The use of the yeast *C. albicans* is of particular interest because it is recognized as an important pathogen in nosocomial infections [50]. Also DCS1 lipopeptides could be considered as a good alternative for controlling the growth of biofilms of *S. typhimyrium*, *S. aureus* and *B. cereus* which are responsible for opportunistic food-borne illness.

## Conclusion

The current study demonstrated that lipopeptides synthesized by *B. methylotrophicus* DCS1 strain exerted considerable antioxidant action involving several antioxidant mechanisms, including metal ion chelating, hydrogen or electron donation and radical scavenging during peroxidation. Further, the DCS1 lipopeptides showed antimicrobial activities against several microorganisms tested. In addition, they showed anti-adhesive activity against biofilm formation as well as their potential to disrupt pre-formed biofilm. The results obtained suggest the possible use of DCS1 lipopeptides as a potential antioxidant and antimicrobial as well as anti-adhesive agent to reduce microbial adhesion and biofilm formation in biomedical field and food industry.

## Abbreviations

BHA: Butylhydroxyanisol
CFU: Colony-forming units
CV: Crystal violet
DPPH: 2, 2-diphenyl-1-picrylhydrazyl
ED_50_: Effective dose
EDTA: Ethylene diamine tetra acetic acid
FT-IR: Fourier transform infrared spectroscopy
IC_50_: Half maximal inhibitory concentration
LB: Luria-Bertani media
MDA: Malondialdehyde
OD: Optical density
PBS: Phosphate-buffered saline
rDNA: Ribosomal Deoxyribonucleic acid
RNA: Ribonucleic acid
TBARS: Thiobarbituric acid reactive substances
THF: Tetrahydro-furan
TLC: Thin layer chromatography
